# *Stenotrophomonas maltophilia* outbreak in a university hospital: epidemiological investigation and literature review of an emerging healthcare-associated infection

**DOI:** 10.1590/S1678-9946202466046

**Published:** 2024-07-29

**Authors:** Mehmet Erinmez, Feyza Nur Aşkın, Yasemin Zer

**Affiliations:** 1 Gaziantep University, Faculty of Medicine, Department of Medical Microbiology, Gaziantep, Turkey

**Keywords:** Stenotrophomonas maltophilia, Outbreak, Nosocomial infection, Bacteremia

## Abstract

*Stenotrophomonas maltophilia* was considered to be a low-virulence organism. But it has emerged as a prominent opportunistic pathogen in patients with certain risk factors. This study aimed to describe an outbreak experienced in our hospital with all dynamics while evaluating previous *S. maltophilia* outbreak reports. *S. maltophilia* isolates were obtained from a university hospital in Türkiye in a seven-months period. Antimicrobial resistance, type of infections, predisposing factors of infected patients, antibiotic therapy, outcome of infections, and outbreak source were investigated. Also, *S. maltophilia* outbreaks in the literature were reviewed. In the 12 months prior to the outbreak, prevalence rate of clinical samples including *S. maltophilia* was 7/1,000 patient per day, opposed to 113/1,000 patient per day during the outbreak. Although a large number of cases were observed in a short seven-month period, a source of contamination could not be detected. Stable mortality rates (or remaining close to the average) during outbreaks can be attributed to the careful attention paid by laboratory and clinic physicians during procedures. *S. maltophilia* has potential to spread outbreaks and infect patients in operating rooms and intensive care units during invasive procedures.

## INTRODUCTION

*Stenotrophomonas maltophilia* is a glucose nonfermenting Gram-negative bacteria found widely distributed in natural and artificial settings^1^. *S. maltophilia*, traditionally considered to be a low-virulence organism, has emerged as a prominent opportunistic pathogen that causes serious human infections, especially in severely debilitated patients^2^. Bloodstream infections, bone and joint infections, urinary tract infections, endocarditis, pneumonia, and meningitis are among the infections caused by *S. maltophilia* ^1,3,4^. Many fomites and medical equipment in clinical settings may serve as promising reservoirs of *S. maltophilia* infection due to their ubiquitous nature and capability to form biofilm on any type of moist surface^4^. The well-known risk factors for *S. maltophilia* infections include: longer hospital stays requiring invasive procedures, admission to an intensive care unit, organ transplantation, mechanical ventilation, indwelling catheters, prior exposure to antibiotics or immunosuppressant therapy, cystic fibrosis, underlying malignancy, and HIV infection^5^. Aminoglycosides and routinely used carbapenems cannot be used against *S. maltophilia* due to its intrinsic resistance^2^. For its favorable susceptibility and positive clinical results in treated individuals, trimethoprim-sulfamethoxazole (TMP-SMX) is a first-line therapy for *S. maltophilia* infections^6^. This report describes the results of the investigation on the *S. maltophilia* outbreak and aims to evaluate outbreak and patient characteristics.

## MATERIALS AND METHODS

This ambidirectional observational study describes the investigation of *S. maltophilia* isolates obtained from a university hospital in Turkiye in a seven-months period (August 2021 to February 2022). We observed a high isolation rate from various clinical specimens in the different wards and ICUs of a university hospital. The introduction of outbreak screening and control measures in 2021 allowed us to assess the outbreak dynamics. The study investigated TMP-SMX resistance, whether isolates were infective or colonizers, the type of infections, the predisposing factors of infected patients, antibiotic therapy, and the outcome of infections, prognostic factors associated with mortality, and outbreak source. To expose the clinical characteristics of *S. maltophilia* infections in clinical settings, we searched the literature on *S. maltophilia* outbreaks and summarized the data along with the previous reports. For the literature search, the PubMed and Google Scholar databases were explored. The terms “*Stenotrophomonas maltophilia* outbreak”, “*Stenotrophomonas maltophilia* epidemic”, and “*Stenotrophomonas maltophilia* bacteremia” were searched. The reference lists of the articles under investigation have been perused for any overlooked articles and pseudo-outbreaks were excluded.

### Ethical approval

The study was carried out in compliance with the Declaration of Helsinki and approved by the Gaziantep University Ethics Committee for Clinical Trials and by the Gaziantep University Clinical Research Ethics Committee with the consent Nº 2021/161.

### Bacterial isolates

A total of 113 consecutive nonduplicate *S. maltophilia* isolates were obtained from different specimens. Only one isolate was obtained from each patient. For blood culture analysis, the BACTEC FX system (Becton Dickinson, USA) was used. Bacterial identification and antibiotic susceptibility testing were done using the automatic VITEK2 (bioMérieux, France) system. Clinical breakpoints set by the European Committee on Antimicrobial Susceptibility Testing (EUCAST) were used to assess drug susceptibility^7^.

### Patient characteristics

A medical chart review was performed for data on patients’ clinical backgrounds, admission wards, time (days) from admission to the occurrence of *S. maltophilia* infection, underlying diseases, primary focus of infection, results of blood culture, history of antibiotics use (within 30 days before the onset of *S. maltophilia* infection), and prognosis. The source of the infection was clinically determined by referencing the results of microbiological examinations. When some bacteria other than *S. maltophilia* were detected in cultures, it was regarded as a polymicrobial infection. The 30-day and 90-day mortalities were defined as the periods from the onset of *S. maltophilia* infection to patient death. Infection or colonization was distinguished according to clinical diagnoses given in final reports. All *S. maltophilia* strains isolated from sterile sites such as blood and cerebrospinal fluid, as well as those isolated from the skin, mucus membranes, wounds, and endotracheal tubes in the presence of clinical signs and symptoms were considered infections and included in the study. Selected cases were discussed with the physician to reveal the clinical relevance of isolates. The definitive diagnosis of infection was clinically established. Colonization was defined as the presence of *S. maltophilia* on skin, mucous membranes, in wounds, or excretions/secretions without causing adverse clinical signs or symptoms.

### Epidemiological and environmental investigations and control measures

Admission and weekly screening by throat swabs were introduced in September 2021 after recognizing the *S. maltophilia* outbreak in ICUs. Admission screening showed that outbreak strains were acquired in the ICU, a questionnaire was applied to cover risk factors such as bed space, mechanical ventilation, surgery, and bronchoscopy. A total of 76 samples were obtained from different places in the hospital environment such as floor, bed rails, bedside table, ventilator, and intravenous pump.

Intervention techniques were addressed between the infection control unit, the physicians, and the staff throughout the outbreak. To lower the risk of nosocomial transmission, the infection control unit delivered training that emphasized proper hand hygiene. The frequency of water system disinfection was increased and microbiologic testing of pipes were performed. Since most patients were under mechanical ventilation, respiratory therapy was thoroughly examined, focusing on the equipment cleaning, disinfection, suctioning, and treatment delivery. In patient rooms, disposable aprons were implemented. The authorization for ICU access was modified and restricted. A general sanitation program using sodium hypochlorite solution was applied to all fomites in patient rooms.

## RESULTS

A total of 113 isolates were obtained from nonrepetitive patients. However, 42 patients were not included in the study due to colonization or contamination when clinical data were considered. Ages of the 71 individuals that were included in the study ranged from 1 to 97, median age was 53. Of the 71 patients, 65% (n = 46) were hospitalized in the ICU, and 35% (n = 25) were under inpatient care in other wards. The distribution of ICU patients consisted of Anesthesia ICU (n = 12; 17%), Neurology ICU (n = 11; 15%), Internal Medicine ICU (n = 10; 14%), Surgical ICU (n = 7; 10%), and Pediatric ICU (n = 6; 8%). Table 1 shows the clinical and demographic characteristics of the 71 patients included in the study.

**Table 1:** Clinical and demographic characteristics of patients with *S. maltophilia* infection.

	Patient n (%)
Sex	
Male	47 (66.2%)
Female	24 (33.8%)
Age	
< 18	18 (25.3%)
18–65	29 (40.9%)
> 65	24 (33.8%)
Comorbidities	
Malignancy	18 (25.3%)
Diabetes Mellitus	8 (11.2%)
Organ transplantation	1 (1.4%)
Stroke	10 (14.1%)
Neutropenia	8 (11.2%)
Other	26 (36.6%)
Site of infection	
Bloodstream	50 (70.4%)
Respiratory system	16 (22.5%)
CNS	4 (5.6%)
Urine	1 (1.4%)
Patients admitted to an ICU	46 (64.7%)
Patients requiring mechanical ventilation	30 (42.2%)
History of antibiotic use	54 (76%)
Hospital days to SM infection (Mean)	13.29
30-day mortality	4 (5.6%)
90-day mortality	3 (4.2%)
Outcome	
Recovery	64 (90.1%)
Death	7 (9.9%)

CNS = central nervous system; ICU = intensive care unit; SM = *Stenotrophomonas maltophilia*.

Among the specimens analyzed, blood cultures constituted the most prevalent category, accounting for 50 (70%) of the samples, followed by tracheal aspirate cultures (n = 9; 13%), sputum cultures (n = 7; 10%), cerebrospinal fluid (CSF) cultures (n = 4; 6%), and urine cultures (n = 1; 1%). Cases presenting blood culture growth were considered primary bacteremia, since a different primary focus was not detected. Bacteremia occurred in four patients after a cardiovascular interventional procedure. Patients with primary bacteremia who required mechanical ventilation in the ICU made up the entire group of patients who passed away (n = 7; 9.9%). Ventriculoperitoneal shunt was present in all four patients with growth in CSF. Additionally, seven of the patients who had *S. maltophilia* growth in respiratory tract samples also showed polymicrobial growth. *Serratia marcescens* (n = 3), *Pseudomonas aeruginosa* (n = 2), *Acinetobacter baumannii* (n = 2), and *Klebsiella pneumoniae* (n = 1) were accompanying microorganisms. Only two isolates were not susceptible to TMP-SMX (97%). Furthermore, susceptibility results for levofloxacin in the 71 strains included were found to be 4.2% susceptible, 16.9% intermediate susceptible, and 78.9% resistant, whilst those for ceftazidime were found to be 61.9% susceptible, 33.9% intermediate susceptible, and 4.2% resistant.

**Figure 1:**
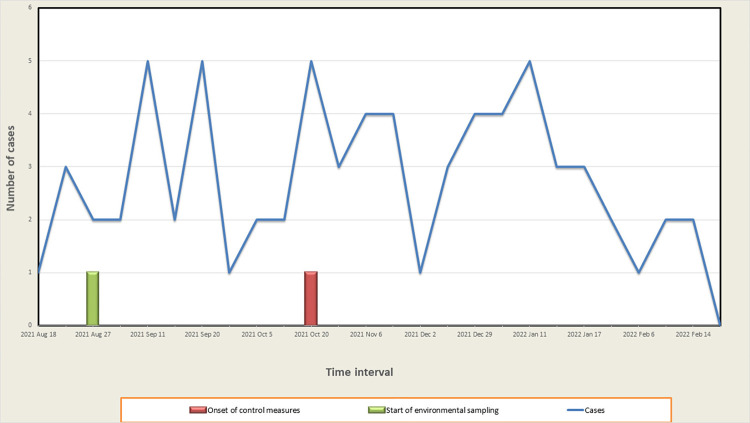
Distribution of *Stenotrophomonas maltophilia* cases from 2021 to 2022.

In the 12 months prior to the outbreak, the prevalence rate of clinical samples including *S. maltophilia* was 7/1,000 patient per days, opposed to 113/1000 patient per days during the outbreak. From March 2022 to April 2022, we performed a post-outbreak observation and the microbiological control of water was within the safety standards. After the interventions, the rates of S. maltophilia cases in the hospital decreased from 113/1000 patients to 2/682 patients receiving inpatient care. When analyzing the case distribution from 2021 to 2022 (Figure 1), we concluded that the onset of environmental sampling and control measures had created a decrease in the number of cases. although these effects were temporary.

In our literature review about *S. maltophilia* outbreaks, we found a total of 20 outbreak reports from different regions. Notably, 75% of these epidemics were from the Asian and European continents. In most of these reports, *S. maltophilia* isolation occurred from blood or respiratory system samples, in which mechanical ventilation stood out as an important predisposing factor. Table 2 describes a detailed summary of the data on the outbreak reports^8-27^.

**Table 2:** Clinical characteristics and manifestations of patients with *Stenotrophomonas maltophilia* infection based on the literature review.

Article	Number of cases	Mean age	Focus of infection	Mortality n (%)	Reported comorbidities or risk factors	Environmental source	Country / Year
Rocha *et al*.^8^	21	57*	Bloodstream	4 (%19)	Hemodialysis patients	Patient rooms	Brazil / 2020
Guyot *et al*.^9^	23	61	Respiratory samples, unspecified	0 (0%)	Recent surgery, intubation, mechanical ventilation	Kitchen tap water	United Kingdom / 2013
Kanaujia *et al.* ^10^	6	ND	Bloodstream	0 (0%)	Intubation, mechanical ventilation	Saline used for suction and in the inspiratory circuit	India / 2022
Cruz-Córdova *et al*.^11^	10	ND	Bloodstream, urine	0 (0%)	ND	Patient rooms, faucet, spout	Mexico / 2020
Ali *et al*.^12^	7	20	Wound	0 (0%)	Burn patients	Scissors used in dressing and bathroom shank	Pakistan / 2017
Guy *et al*.^13^	10	52	Respiratory samples	3 (33.3%)	Respiratory diseases, polytrauma, septic shock, cardiac arrest	Nurses’ station, tap water	France / 2016
Labarca *et al*.^14^	8	ND	Bloodstream	2 (25%)	Bone Marrow Transplant	ND	USA / 2000
Klausner *et al*.^15^	3	37	Respiratory samples	3 (100%)	Bone Marrow Transplant and mechanical ventilation	Ventilator tubing reservoir and the overflow bucket	USA / 1999
Thet *et al.* ^16^	3	51	Bloodstream	0 (0%)	Diabetes mellitus and hemodialysis	Valves of the reverse osmosis outlet and reprocessing machine	Brunei / 2019
Horster *et al*.^17^	26	74	In vitreous specimens and corneal swabs	0 (0%)	Cataract surgery, diabetes mellitus	Buffered sodium saline intraocular rinsing solution	Germany / 2009
Alfieri *et al*.^18^	14	69	Respiratory samples, bloodstream	7 (50%)	Mechanical ventilation	Humidifier of the ventilator	Canada / 1999
Verweij *et al*.^19^	5	0^a^	Respiratory samples, bloodstream	1 (20%)	Preterm infancy	Tap water	Netherlands / 1998
Güvenir *et al*.^20^	11	56	Respiratory samples	0 (0%)	ND	ND	Cyprus / 2018
Motamedifar *et al.* ^21^	16	3	Bloodstream	ND	Pulmonary aspiration, metabolism disorders	ND	Iran / 2017
Lanotte *et al*.^22^	16	ND	Respiratory samples, bloodstream, eye, ear	ND	Neurologic and respiratory diseases, intubation, mechanical ventilation	Water tank of the expiratory circuit of a respirator	France / 2003
Sah *et al*.^23^	7	1	Bloodstream	0 (0%)	ND	Hand of one healthcare provider	Nepal / 2018
Sakhnini *et al*.^24^	2	20	Soft tissue	2 (100%)	Acute myeloid leukemia, aplastic anemia	Faucets	Israel / 2002
Cetin *et al*.^25^	11	0^a^	Bloodstream, respiratory samples,	4 (36%)	Premature birth, perinatal asphyxia	ND	Turkiye / 2015
Gulcan *et a*l.^26^	3	0^a^	Bloodstream	2 (66%)	Meconium aspiration, mechanical ventilation	ND	Turkiye/ 2004
Wang *et al*.^27^	2	67	Cerebrospinal fluid	0 (0%)	Intracranial hemorrhage	Neuroendoscopy	China / 2014

* Median age was described; ^a^Preterm infants or newborns.

## DISCUSSION

The opportunistic pathogen *S. maltophilia* has emerged as a significant global threat and its infections have become more frequently reported^2^. Immunocompromised patients with underlying illnesses or those undergoing invasive procedures are typically affected, since *S. maltophilia* can be found on the surfaces of devices, supplies, and equipment in healthcare facilities^28^. Infections frequently progress as a result of the use of broad-spectrum antibiotics, prolonged hospital admissions, ICU stays, mechanical ventilation, indwelling urinary catheters, and the use of equipment that gets direct contact with the respiratory tract^5^. Respiratory tract infections (pneumonia and acute COPD exacerbations), bacteremia, biliary sepsis, infections of the bones and joints, of the urinary tract, and of the soft tissues, endophthalmitis, eye infections (keratitis, scleritis, and dacryocystitis), endocarditis, and meningitis are among the infections associated with *S. maltophilia* ^1,3,9,11,12,17,27^. In our investigation, *S. maltophilia* was primarily isolated from blood samples (70.4%), followed by samples from the respiratory system (22.5%). Based on our literature search, *S. maltophilia* was predominantly isolated from blood samples (41.6%) and respiratory system samples (34.8%) in a total of 20 outbreak reports, involving 204 patients.

Despite the fact that malignancy was the most frequent underlying cause in our study, the risk factors were confirmed by a large percentage of patients requiring mechanical ventilation and being hospitalized in intensive care. Additionally, the history of antibiotic use in the hospital was at a very high level with 76%. However, considering that the average hospital length of stay before *S. maltophilia* infection was 13.2 days, such outcome was expected. Unfortunately, prolonged hospital stays may require increased use of antibiotics. By reviewing the outbreak reports, we found that hemodialysis was a significant factor in many of the published outbreaks and pseudo-outbreaks. Dialysis is an invasive method that often involves the insertion of needles and catheters. These procedures can ultimately introduce pathogens into the bloodstream if not carried out properly^8^. Biofilms frequently discharge bacteria, chemicals, and endotoxins that can pass through the dialysis membrane and cause an infection^29^. Likewise, bone marrow transplantation seemed to be a facilitating factor for *S. maltophilia* infection. An alteration in the gastrointestinal mucosal barriers may lead to *S. maltophilia* bacteremia in patients. A significant deterioration of the host immune system during the pre-engraftment stage, such as severe protracted neutropenia, as well as the selective action of antibiotics, may raise the risk of bacteremia linked with altered mucosal barriers^14^. An outbreak of *S. maltophilia* meningitis caused by central nervous system interventional procedures has previously been reported in the literature^27^. Similarly, in our study, *S. maltophilia* growth was observed in the CSF samples of four patients with ventriculoperitoneal shunts, indicating that *S. maltophila* is a pathogen that should be considered in procedures involving the central nervous system. We also noticed that outbreaks and pseudo-outbreaks of bronchoscopy-related *S. maltophilia* infections were frequently reported. Bronchoscope examinations are performed on a significant number of patients every day, which emphasizes the need for updating current recommendations, promoting proper hygiene practices, and disclosing new concerns to ensure patient safety^30^.

Malignancy, failure to remove central lines, and ineffective antibiotic treatment were risk factors associated with mortality among patients with *S. maltophilia* bacteremia^31^. ICU stay, malignancy, renal disease, and inappropriate antibiotic therapy were risk factors for mortality of patients with nosocomial *S. maltophilia* pneumonia^32^. In our study, the whole group of deceased patients had been diagnosed with primary bacteremia and were on ventilatory support in the ICU. Previously, researchers reported that attributable mortality of *S. maltophilia* ranged 12%–37.5%^33^. Crude mortality rates were found range from 14% to 69% in patients with *S. maltophilia* bacteremia^4^. For the 71 patients included in our study, the overall mortality rate was 9.9%. Our literature research indicated that among a total of 204 cases in the 20 outbreak reports, the mortality rate was found to be 13.7%, despite the fact that it ranged from 0% to 100% in those reports. We believe that the fact that clinics and laboratories physicians pay closer attention to the protocols during outbreaks explains mortality not rising and remaining near the average. Also, in our study, the TMP-SMX resistance rate was only 3%. A recent meta-analysis of prevalence studies demonstrated that the TMP-SMX resistance rate was 9% worldwide, and that Asia was the most predominant location with a 19.2% rate^34^. However, in the literature, there are reports that define a higher prevalence of TMP-SMX resistance up to 32.8%^35^.

Numerous nosocomial sources, including the hands of medical staff, ventilator circuits, dialysis machines, shower heads, sink traps, and water faucets have yielded *S. maltophilia* isolates^36^. In a number of countries, hospital water systems and contaminated medical equipment hold accountable for most cases of nosocomial *S. maltophilia* infections^37^. Several virulence factors produced by *S. maltophilia* aid in the spread of infection^38^. Those traits have made *S. maltophilia* a significant pathogen attributed to healthcare-associated infections^28^. Hospital outbreaks have been linked to *S. maltophilia* strains that can attach to and form biofilms on medical equipment such as prosthetic equipment, blood, and urine catheters. Antibiotics and immune system defenses are overwhelmed by biofilms^39^. Biofilm formation is facilitated by microbial contamination, presence of organic nutrients, dead ends, low fluxes, and periods of no flow, therefore it is crucial to stop a biofilm from forming at first^29^. Biofilms on faucets, shower heads, and surrounding pipes can be detected via procedures, such as6-monthly pre-flush water samples^9^. In light of the data from previous studies, water systems were the primary area of focus for our investigation into the origins of the outbreak. However, despite intensive environmental sampling and a large number of cases in a short period of seven months, a source of contamination such as tap water, etc., that would cause infection could not be detected. When we examined the epidemic reports on the literature, we found that in 60% of the cases, *S. maltophilia* was isolated from areas with water and water contact, including taps and devices taken together. The organoleptic quality of mains water is improved by carbon filters installed at drinking water dispensers due to the removal of chlorine and other ions. The 6 mm diameter PVC tubes allowed *S. maltophilia* to form a biofilm since chlorine dioxide—a disinfectant—absent after the carbon filter^9^. Interventions include design modifications, such as avoiding dead legs, blind ends, flow straighteners, and unneeded thermostatic mixing valves, as well as removing underutilized outlets and flexible tubes^9^. It is also advised that any carbon filters or narrow-diameter tubing attached to water additions in larger healthcare settings be periodically examined for the growth of nonfermenting microorganisms^9^. In most cases, the outbreak seems to have diminished after better general sterilization protocols were applied^11,14,16,18^. In our study, we observed that, although the number of cases had decreased temporarily as a result of the implementation of environmental sampling and control measures, the outbreak had not halted. It was suggested that the piping system was contaminated by microbial biofilm, due to the persistence of *S. maltophilia* even after the implementation of standard measures to manage the outbreak^8^. Therefore, the researchers concluded that the repair and replacement of deteriorating water system components was beneficial for managing *S. maltophilia* outbreaks^9,11,16^. Also, handwashing measures of the hospital staff have been identified in several studies as a critical component in stopping the outbreak^18,19^.

Hospitals worldwide perform surveillance on infections due to *S. maltophilia* ^4^. Although outbreaks of this species have been reported to date in almost every continent, the cases concentrated especially in the Mediterranean, South Asia, and South America raise the suspicion that hot and humid weather may be related to *S. maltophilia* infections. Increased cell growth rates and concentrations that can come into touch with vulnerable people and potentially raise infection risk are predicted to follow an increase in the global temperature^4^. Likewise, climate change may have an effect on the spread of the *S. maltophilia* infection by causing the spread of waterborne infectious diseases and undermining the value of sanitation in providing clean water supplies^40^. Although, *S. maltophilia* is a typical water bacterium, we were unable to find any additional evidence in the literature to support this assumption.

## CONCLUSIONS

The primary limitation of our investigation was the lack of genetic characterization of the strains due to economic reasons, in order to demonstrate that the same clone probably infected all patients. However, we acknowledge that *S. maltophilia* outbreaks are a very difficult ongoing problem and that some genetic modifications are reasonable to expect. Evaluation of outbreak reports will facilitate the identification of particular risk factors for a specific pathogen. *S. maltophilia* has to be given more consideration as it has the potential to spread outbreaks and infect patients in operating rooms and intensive care units during invasive procedures. Also, it is critical to proceed with the greatest caution when treating burn and bone marrow transplant patients, and patients undergoing hemodialysis and bronchoscopy. The staff members should be regularly observed and trained, and appropriate disinfection protocols should be strictly followed.

## References

[1] Looney WJ, Narita M, Mühlemann K (2009). Stenotrophomonas maltophilia: an emerging opportunist human pathogen. Lancet Infect Dis.

[2] Singhal L, Kaur P, Gautam V (2017). Stenotrophomonas maltophilia: from trivial to grievous. Indian J Med Microbiol.

[3] Falagas ME, Valkimadi PE, Huang YT, Matthaiou DK, Hsueh PR (2008). Therapeutic options for Stenotrophomonas maltophilia infections beyond co-trimoxazole: a systematic review. J Antimicrob Chemother.

[4] Brooke JS (2012). Stenotrophomonas maltophilia: an emerging global opportunistic pathogen. Clin Microbiol Rev.

[5] Al-Anazi KA, Al-Jasser AM (2014). Infections caused by Stenotrophomonas maltophilia in recipients of hematopoietic stem cell transplantation. Front Oncol.

[6] Vartivarian S, Anaissie E, Bodey G, Sprigg H, Rolston K (1994). A changing pattern of susceptibility of Xanthomonas maltophilia to antimicrobial agents: implications for therapy. Antimicrob Agents Chemother.

[7] European Committee on Antimicrobial Susceptibility Testing Breakpoint tables for interpretation of MICs and zone diameters: version 11.0, valid from 2021-01-01.

[8] Rocha VF, Cavalcanti TP, Azevedo J, Leal HF, Oliveira Silva GE, Malheiros AR (2020). Outbreak of Stenotrophomonas maltophilia and Burkholderia cepacia bloodstream infections at a hemodialysis center. Am J Trop Med Hyg.

[9] Guyot A, Turton JF, Garner D (2013). Outbreak of Stenotrophomonas maltophilia on an intensive care unit. J Hosp Infect.

[10] Kanaujia R, Bandyopadhyay A, Biswal M, Sahni N, Kaur K, Vig S (2022). Colonization of the central venous catheter by Stenotrophomonas maltophilia in an ICU setting: an impending outbreak managed in time. Am J Infect Control.

[11] Cruz-Córdova A, Mancilla-Rojano J, Luna-Pineda VM, Escalona-Venegas G, Cázares-Domínguez V, Ormsby C (2020). Molecular epidemiology, antibiotic resistance, and virulence traits of Stenotrophomonas maltophilia strains associated with an outbreak in a Mexican tertiary care hospital. Front Cell Infect Microbiol.

[12] Ali U, Abbasi SA, Kaleem F, Aftab I, Butt T (2017). Outbreak of extensively drug resistant Stenotrophomonas maltophilia in burn unit. J Ayub Med Coll Abbottabad.

[13] Guy M, Vanhems P, Dananché C, Perraud M, Regard A, Hulin M (2016). Outbreak of pulmonary Pseudomonas aeruginosa and Stenotrophomonas maltophilia infections related to contaminated bronchoscope suction valves, Lyon, France, 2014. Euro Surveill.

[14] Labarca JA, Leber AL, Kern VL, Territo MC, Brankovic LE, Bruckner DA (2000). Outbreak of Stenotrophomonas maltophilia bacteremia in allogenic bone marrow transplant patients: role of severe neutropenia and mucositis. Clin Infect Dis.

[15] Klausner JD, Zukerman C, Limaye AP, Corey L (1999). Outbreak of Stenotrophomonas maltophilia bacteremia among patients undergoing bone marrow transplantation: association with faulty replacement of handwashing soap. Infect Control Hosp Epidemiol.

[16] Thet MK, Pelobello ML, Das M, Alhaji MM, Chong VH, Khalil MA (2019). Outbreak of nonfermentative Gram-negative bacteria (Ralstonia pickettii and Stenotrophomonas maltophilia) in a hemodialysis center. Hemodial Int.

[17] Horster S, Bader L, Seybold U, Eschler I, Riedel KG, Bogner JR (2009). Stenotrophomonas maltophilia induced post-cataract-surgery endophthalmitis: outbreak investigation and clinical courses of 26 patients. Infection.

[18] Alfieri N, Ramotar K, Armstrong P, Spornitz ME, Ross G, Winnick J (1999). Two consecutive outbreaks of Stenotrophomonas maltophilia (Xanthomonas maltophilia) in an intensive-care unit defined by restriction fragment-length polymorphism typing. Infect Control Hosp Epidemiol.

[19] Verweij PE, Meis JF, Christmann V, Van der Bor M, Melchers WJ, Hilderink BG (1998). Nosocomial outbreak of colonization and infection with Stenotrophomonas maltophilia in preterm infants associated with contaminated tap water. Epidemiol Infect.

[20] Güvenir M, Otlu B, Tunc E, Aktas E, Suer K (2018). High genetic diversity among Stenotrophomonas maltophilia isolates from single hospital: nosocomial outbreaks or genotypic profile changes during subcultures. Malays J Med Sci.

[21] Motamedifar M, Heidari H, Yasemi M, Sedigh Ebrahim-Saraie H (2017). Molecular epidemiology and characteristics of 16 cases with Stenotrophomonas maltophilia bacteraemia in pediatric Intensive Care Units. Ann Ig.

[22] Lanotte P, Cantagrel S, Mereghetti L, Marchand S, Van der Mee N, Besnier JM (2003). Spread of Stenotrophomonas maltophilia colonization in a pediatric intensive care unit detected by monitoring tracheal bacterial carriage and molecular typing. Clin Microbiol Infect.

[23] Sah R, Siwakoti S, Baral R, Rajbhandari RS, Khanal B (2018). Stenotrophomonas maltophilia causing blood stream infection in neonates and infants: a cause for concern. Trop Doct.

[24] Sakhnini E, Weissmann A, Oren I (2002). Fulminant Stenotrophomonas maltophilia soft tissue infection in immunocompromised patients: an outbreak transmitted via tap water. Am J Med Sci.

[25] Çetin BS, Çelebi S, Özkan H, Köksal N, Sali E, Çelik T (2015). Yenidogan yogun bakim ünitesinde stenotrophomonas maltophilia salgini ve salgin yönetimi. Cocuk Enfeksiyon Dergisi.

[26] Gulcan H, Kuzucu C, Durmaz R (2004). Nosocomial Stenotrophomonas maltophilia cross-infection: three cases in newborns. Am J Infect Control.

[27] Wang CH, Hsu SW, Tsai TH, Wang NC (2014). An Outbreak of trimethoprim/sulfamethoxazole-resistant stenotrophomonas maltophilia meningitis associated with neuroendoscopy. J Med Sci.

[28] De Mauri A, Torreggiani M, Chiarinotti D, Andreoni S, Molinari G, De Leo M (2014). Stenotrophomonas maltophilia: an emerging pathogen in dialysis units. J Med Microbiol.

[29] Coulliette AD, Arduino MJ (2013). Hemodialysis and water quality. Semin Dial.

[30] Mohan A, Madan K, Hadda V, Tiwari P, Mittal S, Guleria R (2019). Guidelines for diagnostic flexible bronchoscopy in adults: joint Indian Chest Society/National College of chest physicians (I)/Indian Association for Bronchology Recommendations. Lung India.

[31] Wu PS, Lu CY, Chang LY, Hsueh PR, Lee PI, Chen JM (2006). Stenotrophomonas maltophilia bacteremia in pediatric patients: a 10-year analysis. J Microbiol Immunol Infect.

[32] Tseng CC, Fang WF, Huang KT, Chang PW, Tu ML, Shiang YP (2009). Risk factors for mortality in patients with nosocomial Stenotrophomonas maltophilia pneumonia. Infect Control Hosp Epidemiol.

[33] Falagas ME, Kastoris AC, Vouloumanou EK, Rafailidis PI, Kapaskelis AM, Dimopoulos G (2009). Attributable mortality of Stenotrophomonas maltophilia infections: a systematic review of the literature. Future Microbiol.

[34] Dadashi M, Hajikhani B, Nazarinejad N, Noorisepehr N, Yazdani S, Hashemi A (2023). Global prevalence and distribution of antibiotic resistance among clinical isolates of Stenotrophomonas maltophilia: a systematic review and meta-analysis. J Glob Antimicrob Resist.

[35] Pien CJ, Kuo HY, Chang SW, Chen PR, Yeh HW, Liu CC (2015). Risk factors for levofloxacin resistance in Stenotrophomonas maltophilia from respiratory tract in a regional hospital. J Microbiol Immunol Infect.

[36] Denton M, Kerr KG (1998). Microbiological and clinical aspects of infection associated with Stenotrophomonas maltophilia. Clin Microbiol Rev.

[37] Cervia SJ, Ortolano AG, Canonica PF (2008). Hospital tap water as a source of Stenotrophomonas maltophilia infection. Clin Infect Dis.

[38] Trifonova A, Strateva T (2019). Stenotrophomonas maltophilia: a low-grade pathogen with numerous virulence factors. Infect Dis (Lond).

[39] Oliveira-Garcia D, Dall'Agnol M, Rosales M, Azzuz AC, Alcántara N, Martinez MB (2003). Fimbriae and adherence of Stenotrophomonas maltophilia to epithelial cells and to abiotic surfaces. Cell Microbiol.

[40] Shuman EK (2010). Global climate change and infectious diseases. N Engl J Med.

